# Molecular Characterization of Potato Virus Y (PVY) Using High-Throughput Sequencing: Constraints on Full Genome Reconstructions Imposed by Mixed Infection Involving Recombinant PVY Strains

**DOI:** 10.3390/plants10040753

**Published:** 2021-04-12

**Authors:** Miroslav Glasa, Richard Hančinský, Katarína Šoltys, Lukáš Predajňa, Jana Tomašechová, Pavol Hauptvogel, Michaela Mrkvová, Daniel Mihálik, Thierry Candresse

**Affiliations:** 1 Biomedical Research Center of the Slovak Academy of Sciences, Institute of Virology, Dúbravská Cesta 9, 84505 Bratislava, Slovakia; Lukas.Predajna@savba.sk (L.P.); Jana.Tomasechova@savba.sk (J.T.); 2 Faculty of Natural Sciences, University of Ss. Cyril and Methodius, Nám. J. Herdu 2, 91701 Trnava, Slovakia; 2795151@student.ucm.sk (R.H.); michaela.mrkvova@ucm.sk (M.M.); daniel.mihalik@ucm.sk (D.M.); 3 Comenius University Science Park, Comenius University in Bratislava, Ilkovičova 8, 84104 Bratislava, Slovakia; katarina.soltys@uniba.sk; 4 Department of Microbiology and Virology, Comenius University in Bratislava, Ilkovičova 6, 84104 Bratislava, Slovakia; 5 National Agricultural and Food Centre, Research Institute of Plant Production, Bratislavská Cesta 122, 92168 Piešťany, Slovakia; pavol.hauptvogel@nppc.sk; 6 INRAE, University of Bordeaux, UMR BFP, 33140 Villenave d’Ornon, France; thierry.candresse@inrae.fr

**Keywords:** genome, next generation sequencing, potyvirus, PVY, Solanaceae

## Abstract

In recent years, high throughput sequencing (HTS) has brought new possibilities to the study of the diversity and complexity of plant viromes. Mixed infection of a single plant with several viruses is frequently observed in such studies. We analyzed the virome of 10 tomato and sweet pepper samples from Slovakia, all showing the presence of potato virus Y (PVY) infection. Most datasets allow the determination of the nearly complete sequence of a single-variant PVY genome, belonging to one of the PVY recombinant strains (N-Wi, NTNa, or NTNb). However, in three to-mato samples (T1, T40, and T62) the presence of N-type and O-type sequences spanning the same genome region was documented, indicative of mixed infections involving different PVY strains variants, hampering the automated assembly of PVY genomes present in the sample. The N- and O-type in silico data were further confirmed by specific RT-PCR assays targeting UTR-P1 and NIa genomic parts. Although full genomes could not be de novo assembled directly in this situation, their deep coverage by relatively long paired reads allowed their manual re-assembly using very stringent mapping parameters. These results highlight the complexity of PVY infection of some host plants and the challenges that can be met when trying to precisely identify the PVY isolates involved in mixed infection.

## 1. Introduction

Plant viruses can be a source of economic loss by causing diseases in economically important plant species. Potyviruses are well known harmful pathogens [1,2], and the genus type species, potato virus Y (PVY) is considered to be one of the most important viruses affecting potato crops [3] and other economically important Solanaceous species (e.g., pepper, tomato, tobacco), as well as weeds and non-solanaceous hosts [1,4]. Furthermore, potyviruses are known to be commonly found in mixed infections, from which synergistic interactions with heterologous viruses are well documented, for example, by inducing more severe symptoms [5].

As the diseases caused by PVY are incurable under field conditions, prophylactic measures are focused on preventing or slowing virus spread in plant populations through the use of resistant varieties [6,7] of healthy controlled propagation material [8] or through the eradication of infected plants. All these phytosanitary actions require access to early, sensitive, and specific detection [9]. Although ELISA and RT-PCR-based techniques provide robust tools for the diagnosis of plant viruses, the large genetic diversity found in many viral species can impose severe constraints on the design and performance of some detection systems [10,11].

To identify a specific virus, molecular detection methods with sufficient sensitivity are usually implemented [12]. Modern diagnostics often require data not only at the level of virus species but they may also require differentiation between viral strains or individual isolates, as these can have different epidemiological properties relevant for the deployment of control strategies [13,14,15]. For example, in the case of PVY, the potato tuber necrotic ringspot disease (PTNRD), which can considerably decrease their market price, or even make the tubers unsellable, is associated with specific recombinant isolates [16,17].

PVY can be described as a complex of strains, among which PVY-C, PVY-N (both PVY-NA-N and PVY-EU-N), and PVY-O represent the nonrecombinant archetypes [17]. However, the PVY-O strain includes a subgroup, PVY-O5, which is serologically and biologically distinct [18]. In addition, the number of recombinant strains is currently known and still, others are being continuously identified [19]. Nine recombinant strains (PVY-N:O, PVY-N-Wi, PVY-NTNa, PVY-NTNb, PVY-NE11, PVY-E, PVY-SYR-I, -II, and -III) are considered as quite common while others (e.g., PVY-N-Wi-156var, PVY-N-Wi-261-4, PVY-SCRI-N) are rare [19,20].

The recombinant strains differ in their genome composition. Although often having a complex mosaic structure, some more or less conservative recombination junction sites have been identified [17,21]. Several RT-PCR-based approaches have been developed to discriminate PVY strains [11,22]. However, to unambiguously assign an isolate to a strain or to identify a divergent variant, a full-length genome characterization is required. The use of high-throughput sequencing (HTS) technologies has brought forth the possibility of unbiased virus detection and identification allowing the gain of a potentially complete view of a plant virome including access to full-length genome data [23,24].

In this work, viromes of 10 tomato and sweet pepper plants were determined from HTS data, enabling us to obtain (nearly) complete genome sequences for several PVY isolates. In some cases, the automated recovery of such full-length genomic sequences was, however, hampered by the occurrence of mixed infections involving different PVY variants.

## 2. Results

### 2.1. PVY Isolates Identified in Non-Potato Hosts Belong to Recombinant Strains

The HTS analysis of nine tomato samples and one sweet pepper sample revealed the presence of PVY and, except for one tomato sample (T31), where only PVY infection was identified, the presence of additional virus(es) (Table 1). 

A single large contig covering the full-length (or nearly full-length) PVY genome was recovered from the HTS dataset for 7 of the 10 samples (T101, T20, T24, T31, PAP, SL50V, PAR-P2) (Table 1). However, due to the presence of other virus(es), the role of PVY in the etiology of the range of symptoms observed (Table 1) remains undetermined.

**Figure 1:**
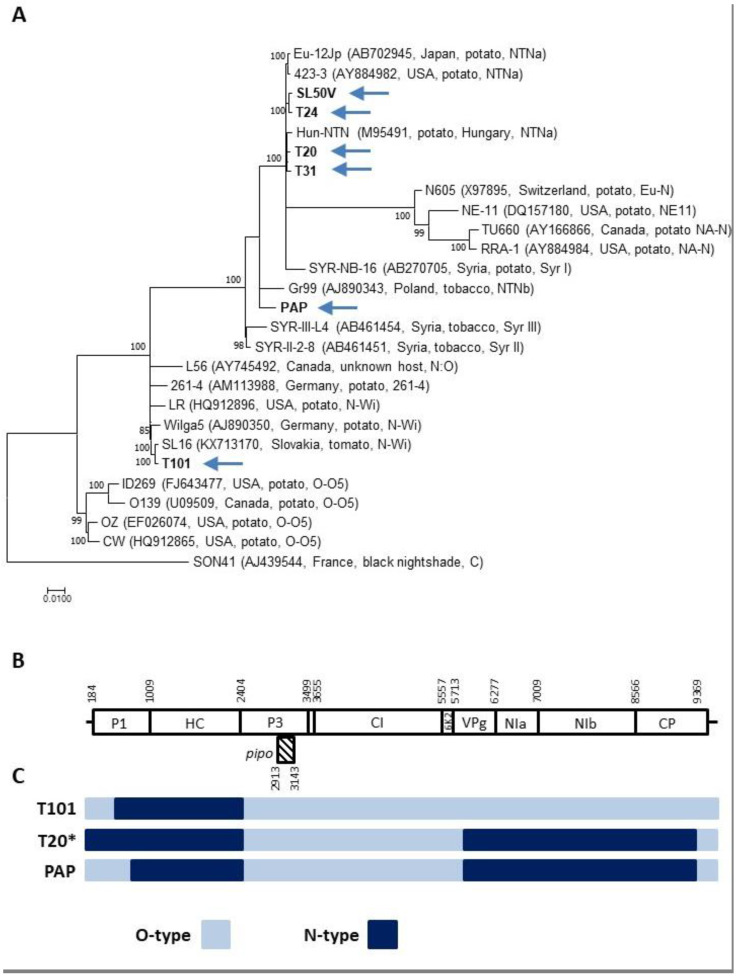
Analysis of samples showing single variant infections by PVY. (**A**) A maximum likelihood (ML) phylogenetic tree showing the relationship among PVY isolates. Complete genomes of PVY isolates determined in this work (highlighted by an arrow), together with sequences of the selected representing isolate belonging to different molecular groups, were used for phylogenetic analysis. The database isolates are identified by their names, GenBank accession number, country of origin, and strain relationship. Strain affiliation is indicated based on [20]. The phylogenetic analysis was inferred using maximum likelihood (ML) based on the General Time Reversible (GTR + G) model selected as the best-fit model of nucleotide substitution based on Bayesian information criterion (BIC) as implemented in MEGA 7. The divergent PVY isolate AJ439544 was used as an outgroup. Scale bar represents genetic distance and the numbers at the nodes indicate the bootstrap values (1000 replicates) >70%. (**B**) Schematic representation of the PVY genome showing the nucleotide positions delimiting the respective potyviral functional products (based on the complete genome of SL16 isolate (KX713170, PVY-N-Wi strain). (**C**) Schematic representation of recombinant PVY genomes isolates characterized in this work, showing the position of parental genome portions. PVY-O-type (azure), PVY-N-type (dark blue). * counts also for the T24, T31, PAP, SL50V, and PAR-P2 genomes.

The multiple alignments and phylogenetic analysis of the obtained sequences, together with representatives of the different PVY strains, assigned the Slovak isolates to three molecular groups. The T101 tomato isolate shows a recombination pattern and strong phylogenetic affinities to the PVY-N-Wi strain. Five isolates from tomato (T20, T24, T31, SL50V, PAR-P2) belong to the PVY-NTNa recombinant strain, while the pepper PAP isolate shares the characteristics of members of the PVY-NTNb strain (Figure 1C, Table 2). 

All the deduced PVY polyprotein sequences were of the same length (3061 aa) and showed all the expected conserved amino acid motifs characteristic of PVY without any obvious peculiarities or originalities.

### 2.2. Mixed Infection Involving Different PVY Isolates

De novo assembly of the T1, T40, and T62 HTS datasets, together with a visual inspection of mapped reads indicated the presence of genetically different PVY variants in the plant sample. To investigate this point, four informative genomic regions (nt 37-493, 704-2407, 3001-4948, and 5880-8806) from reference O-type (accession number U09509) and N-type (AJ585197) isolates, selected based on genome differences (see Karasev and Gray [17]), were used for mapping the reads of the T1, T40, and T62 datasets using stringent parameters (minimum overlap 25, minimum overlap identity 95% in Geneious mapper).

**Figure 2:**
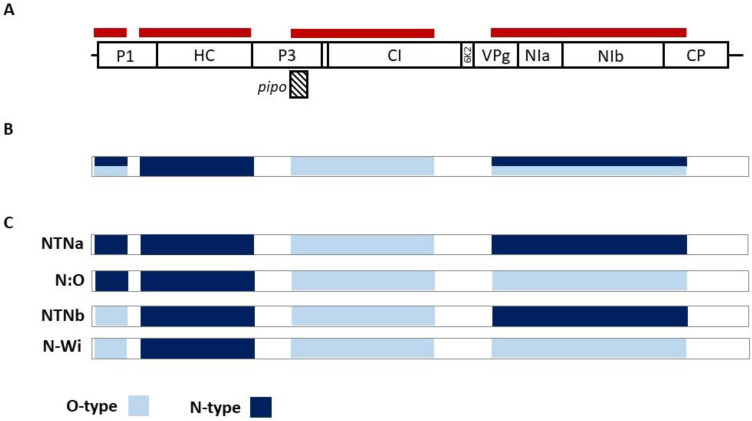
Analysis of samples showing multiple infections by PVY strains. (**A**) Schematic representation of the positions of informative regions (red stripes) along the PVY genome used for PVY-O- and N-specific mapping of HTS reads (**B**) Graphical representation of obtained sequences from samples T1, T40, and T62, where positions with more than one variant detected are shown (azure/dark blue) (**C**) Possible recombination patterns of PVY variants present in T1, T40, and T62 samples.

While reads corresponding to only one of the PVY strains were identified in all three samples in the 704-2407 and 3001-4948 genomic regions, both O- and N-type reads were obtained when analyzing the 37-493 and 5880-8806 genomic parts. These two regions show, respectively, 31.5–32.3% and 14.4–15% nucleotide divergence between the O and N strains (Table 3). Due to the recombination pattern and to the recombination junction sites sharing similarities among the various PVY strains (Figure 2B,C, Table 2), the exact identification of the PVY variants involved in the mixed infections present in the T1, T40, and T62 plants could not be established.

To confirm the presence of O- and N-type sequences covering the genome portions for which reads of both strains were identified (either UTR-P1 or NIa), RT-PCR assays were carried out using specific primers (Table 4). For all three plants, both expected PCR products (N and O-specific) were obtained, confirming the in silico data.

### 2.3. Attempts to Reconstruct Complete PVY Genomic Sequences from Mixed Infection

**Figure 3:**
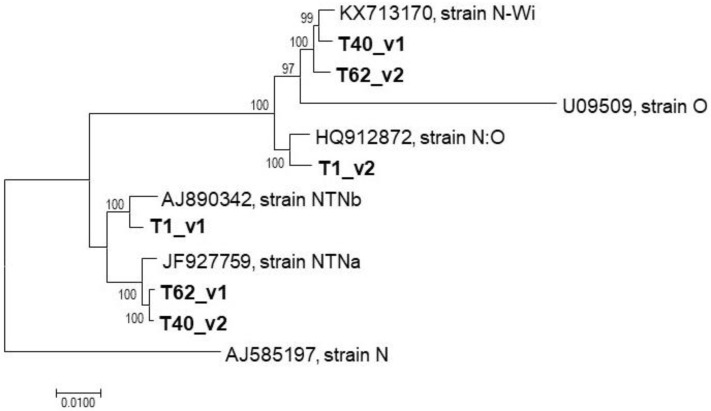
Phylogenetic analysis of PVY variants identified in mixed infections. The phylogenetic tree reconstructed from nearly complete genomes of T1, T40, and T62 variants and selected reference genomes using the neighbor-joining algorithm implemented in MEGA v.7.

Automated assembly of the T1, T40, and T62 HTS dataset did not result in complete genomes (the largest contig obtained represented <75% of a full genome and, with this exception, all other contigs represented less than about half of the full genome size) and did not allow the proper identification of the PVY variants involved. Therefore, a manual reconstruction approach was evaluated, using extremely stringent conditions that could potentially allow the separation of closely related variants. Since it was supposed that long reads would be critical for the reconstruction of closely related haplotypes, a specific trimming step in which only high-quality paired reads (>140 bp) were selected was performed. Tentative scaffolds were manually reconstructed from contig sequences and were then validated and extended by successive rounds of mapping at very high stringency (>95% reads length showing >98% nt identity) using only the long high quality reads selected as described above. This approach allowed for the gradual separation of haplotypes, even in genome regions in which the isolates in mixed infection showed in the order of a few percent of nucleotide divergence. It should be stressed that these reconstructions did not involve any reference sequence information and that the genomes were thus reconstructed without any a priori or fixed expectations on the final outcome. The obtained nearly complete genomes (Genbank Accession numbers MW685827–MW685832) were finally validated by mapping of the selected long high quality paired reads using a full identity criterion (100% identity over 100% of reads length), giving strong confidence in the obtained sequences thanks to their deep coverage (in all cases in excess of 250x average coverage). A phylogenetic analysis of the reconstructed genomes together with reference isolates finally allowed us to unambiguously determine that the T1 was simultaneously infected with NTNb and N:O isolates while the T40 and T62 ones were co-infected by NTNa and N-Wi isolates (Figure 3).

## 3. Discussion

Mixed infection of plants with several viruses belonging to different taxa is now frequently detected, especially thanks to the application of massive parallel sequencing [24,25,26,27,28]. In this work, the analysis of leaf samples from tomato and pepper indeed revealed mixed infections of PVY (a potyvirus) and other viruses (from the Cucumovirus, Carlavirus, and/or Ophiovirus genera, Table 1) further confirming the complexity of the virome that can be present in single plants [29,30]. 

De novo assembly of HTS reads or their mapping to reference viral genomes is standardly used in order to access the infecting viruses’ genome data [9,31,32]. Because of frequent recombination events in PVY evolutionary history, resulting in a genome with mosaic structure involving both PVY-N and PVY-0 parents in the case of most PVY recombinant strains [17,33], such approaches can be challenging. 

Assembly of the HTS reads generated from ribosomal-depleted total RNA (1.5–3.9 M of 93–185 bp length) allowed us to obtain full-length or nearly full-length PVY genomes (i.e., missing only a few nucleotides at the genome extremities) with a high coverage from 7 of the 10 tested samples (Table 1). In all these cases, a single PVY variant was unambiguously identified, belonging to the PVY-N-Wi, PVY-NTNa, or PVY-NTNb strains. These results complement a previous more limited work [34], showing the presence of recombinant PVY strains in Slovakia based on RT-PCR targeting various recombination junction (RJ) sites. 

Recombination clearly played a significant role in the evolutionary history of PVY [10,17,35]. Recombination, together with mutation, increases the genetic variability of the given taxa, which can result in higher viral fitness or survival of the virus population in a previously nonviable environment, sometimes leading to the emergence of new resistance-breaking strains [36,37]. Indeed, a change in the PVY strain prevalence from a non-recombinant to recombinant one during the last few decades was generally observed [6,38,39] suggesting a better adaptation of current recombinant strains over non-recombinant ones. Similarly, the latest reports of PVY strain characterization in infected plants point to a growing incidence of recombinant PVY strains [40]. PVY recombinant strains were previously characterized to be quite conserved regarding the positions of recombination junctions within their genomes [21]. Four RJs seem to be deeply conserved and have very similar or even identical positions between several strains (PVY-N:O, PVY-N-Wi, PVY-NTNa, PVY-NTNb). From these RJs, the first and the last RJ show more variety between rare PVY recombinants, while the second and third RJs are more stably shared [17,19]. Because of this peculiar pattern of variation within the PVY species, it was previously recommended to use sequencing or multiple pairs of specific primers targeting RJs within the PVY genome in order to assign an isolate to a particular strain [11,41]. However, the growing evidence of “divergent” PVY isolates escaping accurate identification using such tests, which were developed with a limited pool of isolates [42], pinpoints the usefulness of complete genome analyses to characterize PVY strain variability [16]. Indeed, the challenges of viral strain identification due to extensive recombination were recently addressed for the sugarcane mosaic virus (SCMV), another member of genus potyvirus, where it was advised to use whole genome phylogenetic analysis for viral strain identification [43].

Mixed infections by different PVY isolates are reported to be quite common [44,45]. In the work reported here, 3 of the 10 tested samples (T1, T40, and T62) were identified as bearing a mix of different PVY variants. As a consequence of these mixed infections, it was not possible to de novo assemble full-length PVY genomes for these three samples. The mixed infection status was evident both from the assembled partial contigs and from the mapping of HTS reads against selected portions of PVY-O and PVY-N genomes, which showed the simultaneous presence of N-type and O-type sequences spanning the same genomic regions (Table 2). These in silico results were further confirmed by N- and O-type specific RT-PCRs. Unfortunately, as a consequence of the conservation of the RJ region in the various PVY recombinant strains and of the sharing of genomic regions between the recombinants involved, it was not possible to automatically reconstruct full-length PVY genomes or haplotypes, even when trying a range of assembly parameters. While the data confirmed the presence of at least two PVY strains in the three samples, it was not possible to precisely determine the identity of the individual strains present in each sample by a standard bioinformatics approach. Indeed, these could potentially be identified as either NTNa and NWi or N:O and NTNb, or different combinations of these strains (Figure 2).

Only manual reassembly making use of long paired reads mapped at very high stringency against manually assembled scaffolds ultimately allowed the reconstruction of near-complete genomic sequences and the identification of the PVY strains involved in these mixed infections. In practice, as soon as two isolates in coinfection share a region of perfect identity that is longer than the reads available, it will not be possible to reconstruct the genomic haplotypes over this region. However, in practice, the various PVY recombinant strains are not identical in their shared genomic regions. The basis for this small divergence is that (i) the various recombinants independently emerged from different parental isolates and (ii) the recombinants have independently evolved and further diverged since their emergence. As a consequence, PVY recombinant strains are not identical in their shared genomic regions but, on the contrary, diverge by a limited percentage, as can clearly be seen in phylogenetic reconstructions focusing on such regions. This limited divergence translates into an average of 1–2(3) mutations/100 nt over the shared regions. Given the average 250 nt covered by the long paired reads used here, this small level of differentiation is sufficient to reconstruct or validate haplotypes over long genomic regions even if shared by co-infecting recombinants. Although this cannot be unambiguously established, we suggest that several factors were critical in the ability to perform this reconstruction including (i) mixed infections that involved only two isolates, (ii) very deep coverage (>250x in all cases), and (iii) availability of long (>140 nt) paired reads (and up to ~600 nt of unread sequence between reads of a pair). The last two points were in particular critical for the ability to separate haplotypes over shared regions and unambiguously reconstruct haplotypes over recombination junctions. 

It should be noted that even though we detected at least two PVY strains in mixed infection from a single leaf sample, one can wonder whether such a state is stable or transient because of possible virus-virus interactions, such as cross protection [46]. 

Several reports of PVY interactions in mixed infections have been previously published, showing an antagonistic nature, depending on the viral strains [13,47], and even partial cross-protection [48]. It was shown that PVY-NTN superinfection decreased the titers of PVY-N:O and PVY-N-Wi, while existing PVY-NTN infection prevented infection by secondary virus in some cases, while its titer was unaffected by PVY-N:O, PVY-N-Wi, or PVY-O [48]. Such information could be useful to discriminate between potential strain mixtures as in the case reported here, although it would be impossible to determine the time of infection by individual strains. 

In this work, mixed infections involving PVY recombinant isolates proved recalcitrant to automated genome reconstruction and strain identification following Illumina HTS. It was however possible to manually perform these tasks. Such a problem with strain identification could be potentially avoided by using single molecule long sequencing reads such as those generated by MinION (Oxford Nanopore) as proposed by Pooggin [49] (2018). Indeed, Della Bartola et al. [45] (2020) showed that it is possible to assemble and differentiate individual PVY strains in mixed infections using MinION, while it was not possible to individuate strains using specific primer pairs which otherwise worked well for most samples, supporting the former proposal.

## 4. Materials and Methods

### 4.1. Plant Samples

Nine tomato (*Solanum lycopersicum* L.) plants and one sweet pepper (*Capsicum annum* L.) plant were sampled during their vegetation period (July-September 2017–2019) and analyzed for the presence of viruses using HTS (Table 1). All the samples originated from plants grown in home gardens in Slovakia.

### 4.2. HTS Analysis

Total RNAs were extracted from a single leaf from the upper part of plants (ca 0.2 g) using the Spectrum Plant Total RNA Kit (Sigma Aldrich, St. Louis, MO, USA). Subsequently, ribosomal RNAs were removed using the Ribo-Zero rRNA Removal Kit (Illumina, San Diego, CA, USA). Samples of ribosomal-depleted total RNAs were used for double-stranded cDNA synthesis using the SuperScript II kit (Thermo Fisher Scientific, Waltham, MA, USA). The cDNA was then purified with 2.2x AMPure XP beads and quantified with the Qubit 2.0 Fluorometer (Thermo Fisher Scientific, Waltham, MA, USA). The samples were then processed with the transposon-based chemistry library preparation kit (Nextera XT, Illumina, San Diego, CA, USA). Low-cycle PCR and mutual indexing of the fragments were carried out. Fragments were purified with 1.8x AMPure XP beads (BeckmanCoulter, Brea, CA, USA) without size selection. The fragment size structure of the DNA libraries was assessed using the Agilent 2100 Bioanalyzer (Agilent Technologies, Santa Clara, CA, USA). The equimolar pool of 4nM DNA libraries was denatured, diluted to 10 pM, and sequenced (300-bp paired-end sequencing) on the Illumina MiSeq platform (Illumina, San Diego, CA, USA).

High-quality trimmed reads were used for de novo assembled using a CLC Genomics Workbench 7.5 (https://www.qiagenbioinformatics.com/, accessed on 5 October 2020) with automatic graph parameters set and with reads mapped back to contigs with the following parameters (Mismatch cost 2, Insertion cost 3, Deletion cost 3, Length fraction 0.7 and Similarity fraction 0.9) and minimum contig length 1000 bp. All contigs were subsequently aligned to the viral genomes database (ftp://ftp.ncbi.nih.gov/genomes/Viruses/all.fna.tar.gz, accessed on 5 October 2020).

The reads were again mapped against the obtained full-length or nearly full-length sequences using Geneious v.8.1.9 to confirm, complete or edit their sequence. Genomes of reference PVY isolates (U09509, PVY-O-type) and AJ585197 (PVY-N-type) were retrieved from the Genbank database (www.ncbi.nlm.nih.gov, accessed on 5 October 2020). In the case of three HTS datasets (T1, T40, T62), reads were mapped against four informative portions of the PVY genome (nts 37-493, 704-2407, 3001-4948, and 5880-8806) from U09509 (PVY-O) and AJ585197 (PVY-N) using more stringent parameters implemented in Geneious (minimum overlap 25, minimum overlap identity 95%).

In the case of three samples for which mixed infections involving recombinant isolates did not allow the automated reconstruction of full-length genomes, the contigs obtained were manually assembled into scaffolds which were further extended and validated by successive rounds of reads mapping at extremely high stringency (95% of reads length, 98% identity) using CLC Genomics workbench v21.0.3. Only long (>140 nt), high-quality paired reads selected by a specific trimming step were used in this reconstruction that allowed us to separate coinfecting recombinants even in their shared genomic regions and to reconstruct the complete genomic haplotypes. Final validation of the obtained near-complete genomic sequences was performed by a final mapping of the selected high-quality read pairs at an extreme stringency level (100% identity over 100% of reads length) to ensure the accuracy of the assembled genomes.

Phylogenetic analyses and comparisons were performed using the MEGA v.7 [50] and DnaSP v.5 [51] programs.

### 4.3. Strain Specific RT-PCR Assays

O-type and N-type specific RT-PCR targeting portions of the 5′UTR-P1 and NIa gene (Table 4) were carried out on a template consisting of random hexamer-derived cDNA employing the same RNA isolation as used for the preparation of HTS libraries. For all primer combinations, the following cycling conditions were used: denaturation at 98 °C for 1 min, 35 cycles of amplification (98 °C for 30 s, 53 °C for 30 s, and 72 °C for 30 s), and a final extension at 72 °C for 5 min.

## Figures and Tables

**Table 1:** List of samples used for HTS analysis and their characteristics.

Sample	Natural Host	Locality	Year of Sampling	Symptom on Leaves	Additional Viruses Identified in the Sample ^1^
T101	tomato	Pezinok	2019	leaf narrowing and deformation	CMV
T20	tomato	Paňa	2017	mild leaf distortions	PVM
T24	tomato	Nitra	2017	mosaics	CMV
T31	tomato	Švošov	2017	symptomless	-
PAP	sweet pepper	Čachtice	2019	mosaics	RWMV
SL50V	tomato	Pezinok	2018	curling, mosaics, deformations	CMV
PAR-P2	tomato	Pezinok	2018	Mosaics	LRNV
T1	tomato	Paňa	2017	deformations, vein clearing	CMV
T40	tomato	Plavecký Mikuláš	2017	symptomless	PVM, PVS
T62	tomato	Sološnica	2017	curling	CMV, PVM, PVS

^1^ the near-complete non-PVY viral genomes reconstructed, CMV: Cucumber mosaic virus (RNA1, RNA2, RNA3), LRNV: Lettuce ring necrosis virus (RNA1, RNA2, RNA3), PVM: Potato virus M, PVS: Potato virus S, RWMV: Ranunculus white mottle virus (only partial genome available in Genbank).

**Table 2:** Analysis of HTS data from the samples containing only one detectable PVY sequence variant.

Sample	Number of Reads	Mean Length (bp) of Reads Mapping PVY	Number of Reads Mapped to the Determined Genome	Mean Sequence Depth	Accession Number	The Closest BLAST Relative (% of nt Identity)	PVY Strain ^1^
T101	3,168,840	176.4	46,134	835.9	MW595185	KX713170 (Slovakia, tomato) 99.63%	N-Wi
T20	3,585,846	93.1	846,986	9196.7	MW595182	JF927752 (Poland, tobacco) 99.72%	NTNa
T24	2,215,328	116.7	53,849	670.5	MW595183	KX184818 (Israel, potato) 99.75%	NTNa
T31	2,666,282	129.7	58,843	793.6	MW595184	JF927761 (Poland, tobacco) 99.89%	NTNa
PAP	542,034	149.7	57,741	948.1	MW595181	AB185833 (Syria, potato) 98.61%	NTNb
SL50V	2,550,640	119.4	6075	80.5	MW595187	MH937417 (Germany, potato) 99.81%	NTNa
PAR-P2	3,912,270	185.8	147,409	2830.2	MW595186	KX184817 (Israel, potato) 99.75%	NTNa

^1^—based on Karasev and Gray [17].

**Table 3:** Analysis of HTS data from the samples containing more than one PVY sequence variant. Partial sequences available in the Genbank databases under accession numbers MW595190–MW595207.

			nt 37-493 ^a^		nt 704-2407		nt 3001-4948		nt 5880-8806	
Sample	Number of Reads	Mean Length of Reads Mapping PVY (bp)	O-Type Reads (Sequence Depth)	N-Type Reads (Sequence Depth)	O-Type Reads (Sequence Depth)	N-Type Reads (Sequence Depth)	O-Type Reads (Sequence Depth)	N-Type Reads (Sequence Depth)	O–Type Reads (Sequence Depth)	N-Type Reads (Sequence Depth)
T1	1,855,568	115.3	961 (250.6x)	408 (102.4x)	-	18,488 (1214.5x)	12,684 (705.5x)	-	12,276 (478.1x)	8991 (357.2x)
T40	2,906,680	119.3	877 (242.3x)	2139 (556.2x)	-	41,975 (2811.3x)	43,905 (2547.5x)	-	14,569 (588.5x)	44,405 (1775.4x)
T62	2,886,468	122.1	710 (196.2x)	1179 (316.3x)	-	25,723 (1753.9x)	22,494 (1317.1x)	-	9351 (381.7x)	19,524 (795.4x)

^a^—position of the genomic portion corresponding to the full-length PVY genome (U09509).

**Table 4:** Primer used for specific RT-PCR detection.

Primer	Sequence (5′ → 3′)	Orientation	Genome Portion	Specific Target
PVY-O-127F	GGAAACCATTTCAACTCAAC	+	UTR-P1	O
PVY-O-469-R	CTGGAAGTGATATTCTTCCC	−		O
PVY-N-125F	GTGTAAGCTATCGTAATTCAG	+		N
PVY-N-487R	AACACTTGACGCAGCCATTTG	−		N
PVY-O-6320F	GCCCAAACAGTTTGTAGGCTG	+	NIa	O
PVY-O-6811R	GTAGTTCGTGGTGTGTTTGTTG	−		O
PVY-N-6359F	GGAACGTCTGAAATGTATGGG	+		N
PVY-N-6796R	CACATTATTCGCCAAGCTGTG	−		N

## Data Availability

The nucleotide sequences reported in this paper have been deposited in the GenBank database (www.ncbi.nlm.nih.gov, accessed on 13 April 2021) under the accession numbers listed in the text.
